# Role of chest CT scan in atypical cardiac trauma management: Left ventricle injury by a nail gun

**DOI:** 10.1016/j.radcr.2021.07.068

**Published:** 2021-08-26

**Authors:** Shokoufeh Hajsadeghi, Sam Zeraatian Nejad Davani, Arash Pour Mohammad, Milad Gholizadeh Mesgarha

**Affiliations:** a Research center for prevention of cardiovascular disease, Institute of endocrinology & metabolism, Iran University of Medical Sciences (IUMS), Tehran, Iran; b Department of cardiovascular surgery, Rasool akram hospital, Iran University of Medical Sciences (IUMS), Tehran, Iran; c Faculty of Medicine, Iran University of Medical Sciences (IUMS), Tehran, Iran

**Keywords:** Nail gun injury, Penetrating heart injury, Sternotomy, Wrapping repair

## Abstract

We report a case of an accidental penetrating cardiac trauma with a nail gun. A 28-year-old man was repairing a sofa with a nail gun when a nail was misfired to his chest. At the time of his presentation, he underwent chest CT scan, showing the nail as a sharp hyperdense foreign body penetrating the chest wall passing through the lower lobe of the left lung and finally the anterior aspect of left ventricle cavity. This report highlights the utility of the chest CT scan to detect trajectory of the misfired nail accurately and instantaneously in a hemodynamically stable patient to assist in the surgery plan.

## Introduction

Chest trauma comprising cardiac injury is one of the most common causes of morbidity and mortality giving rise to virtually 25% of trauma-related deaths [1]. The penetrating one is mostly caused by a gunshot wounds and stab wounds but fairly rare its cause can be nail gun injuries with 25% mortality [2,3]. Hence, early diagnosis and then essential surgical intervention are paramount. With the advent of multi-detector CT scan, accurate, and instantaneous understanding of injury location is plausible [1]. Herein, a case of penetrating cardiac trauma with nail gun injury and its early detection with chest CT scan gave aid in surgery plan presented, with the aim of enhancing further use of imaging modality in atypical penetrating cardiac trauma.

## Case report

**Fig. 1:**
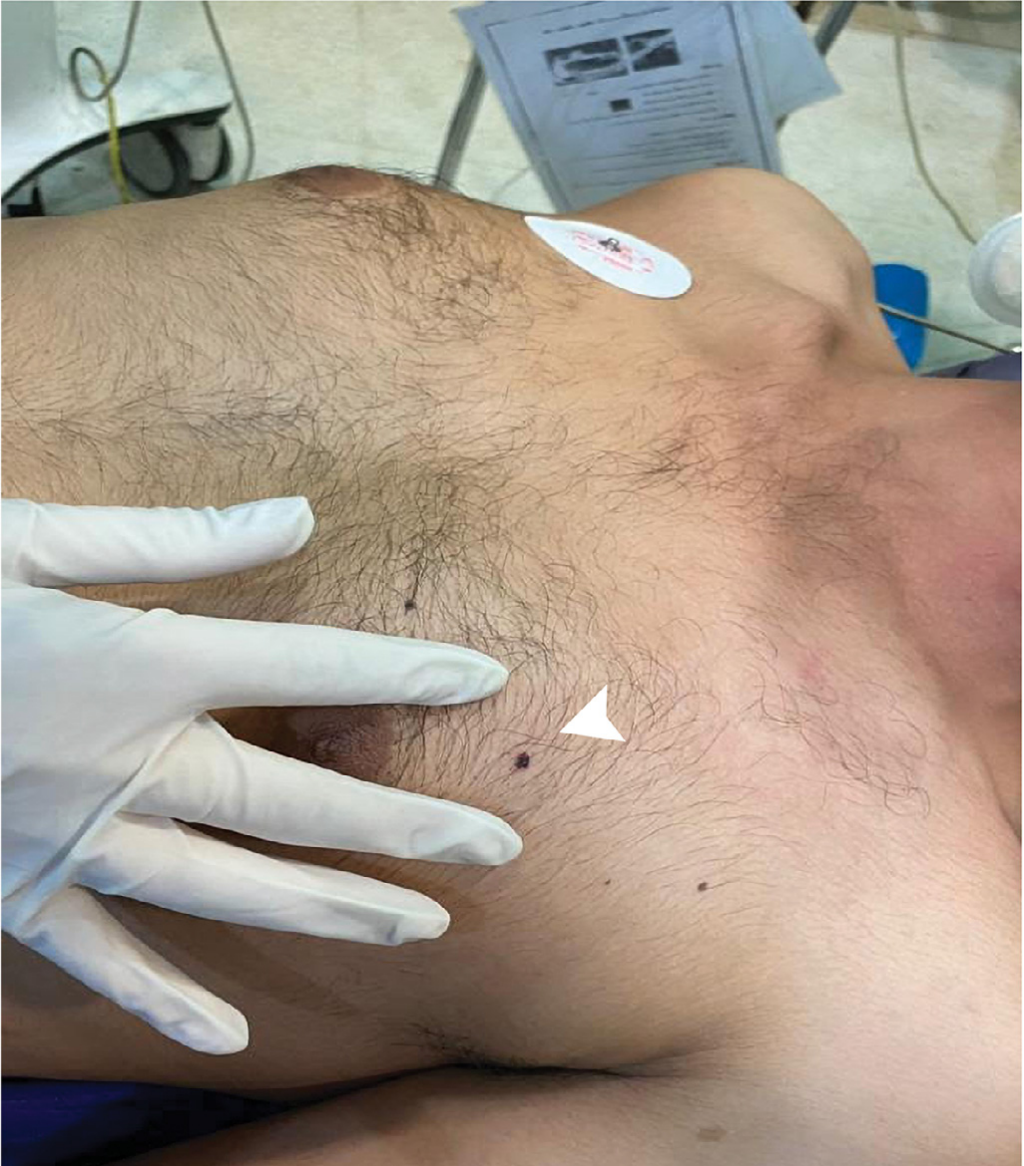
The patient's chest picture showed a clean circular puncture wound in the third intercostal space of the left hemithorax at the midclavicular line (white arrowhead) The nail head stopped at chest wall limiting further penetration.

**Fig. 2:**
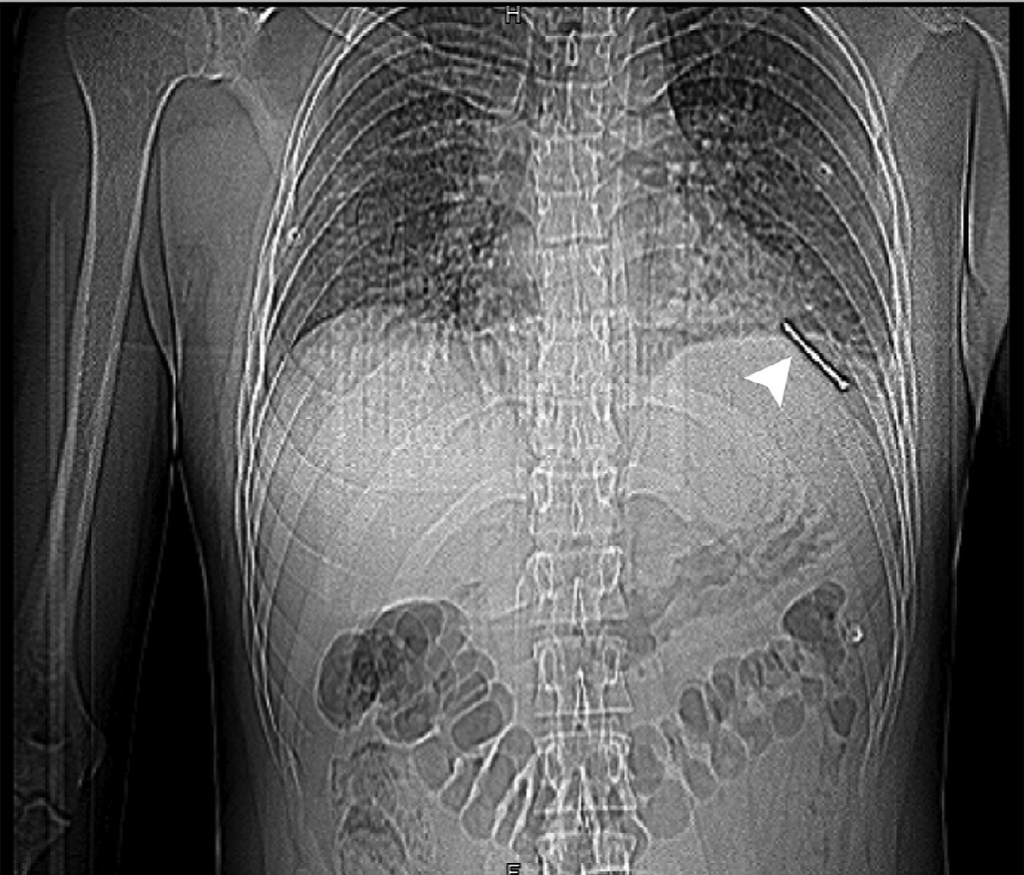
AP scout view of the chest CT showed the whole nail route in the chest (white arrowhead).

**Fig. 3:**
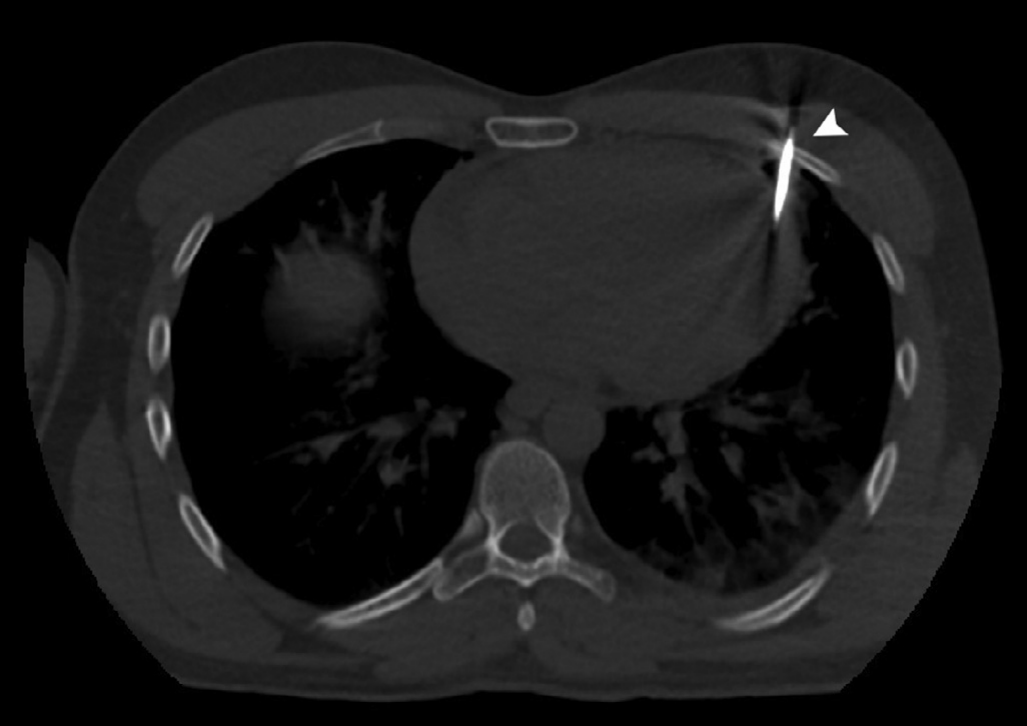
Mediastinal window of the chest CT demonstrated left ventricle wall and cavity penetration by nail (white arrowhead).

**Fig. 4:**
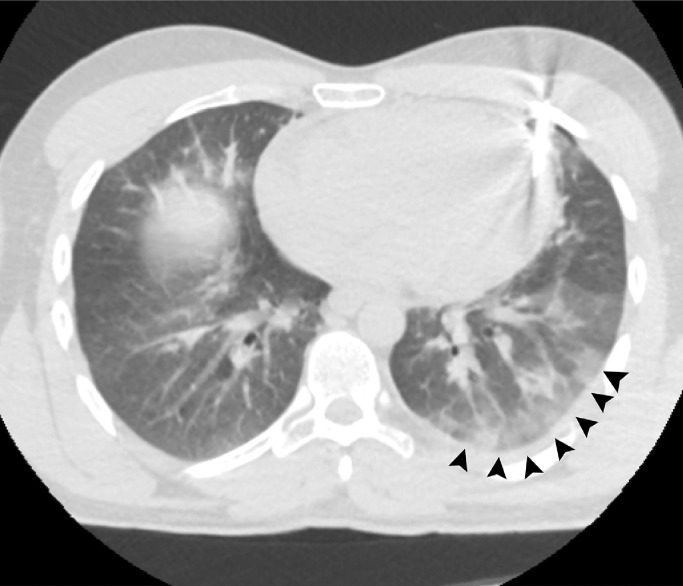
Lung window of the chest CT demonstrated subsequent pulmonary hemorrhage (black arrowheads).

**Fig. 5:**
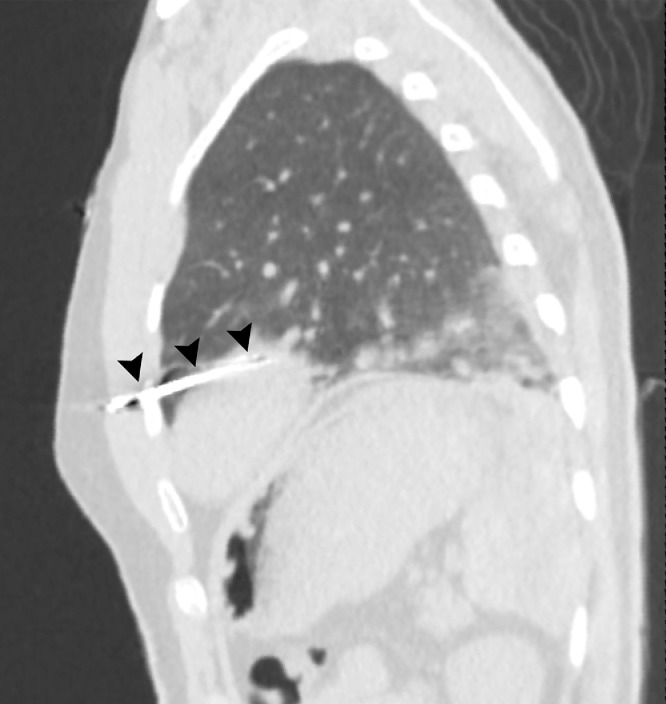
Sagittal view of the chest CT-lung window, the nail is shown (black arrowheads).

**Fig. 6:**
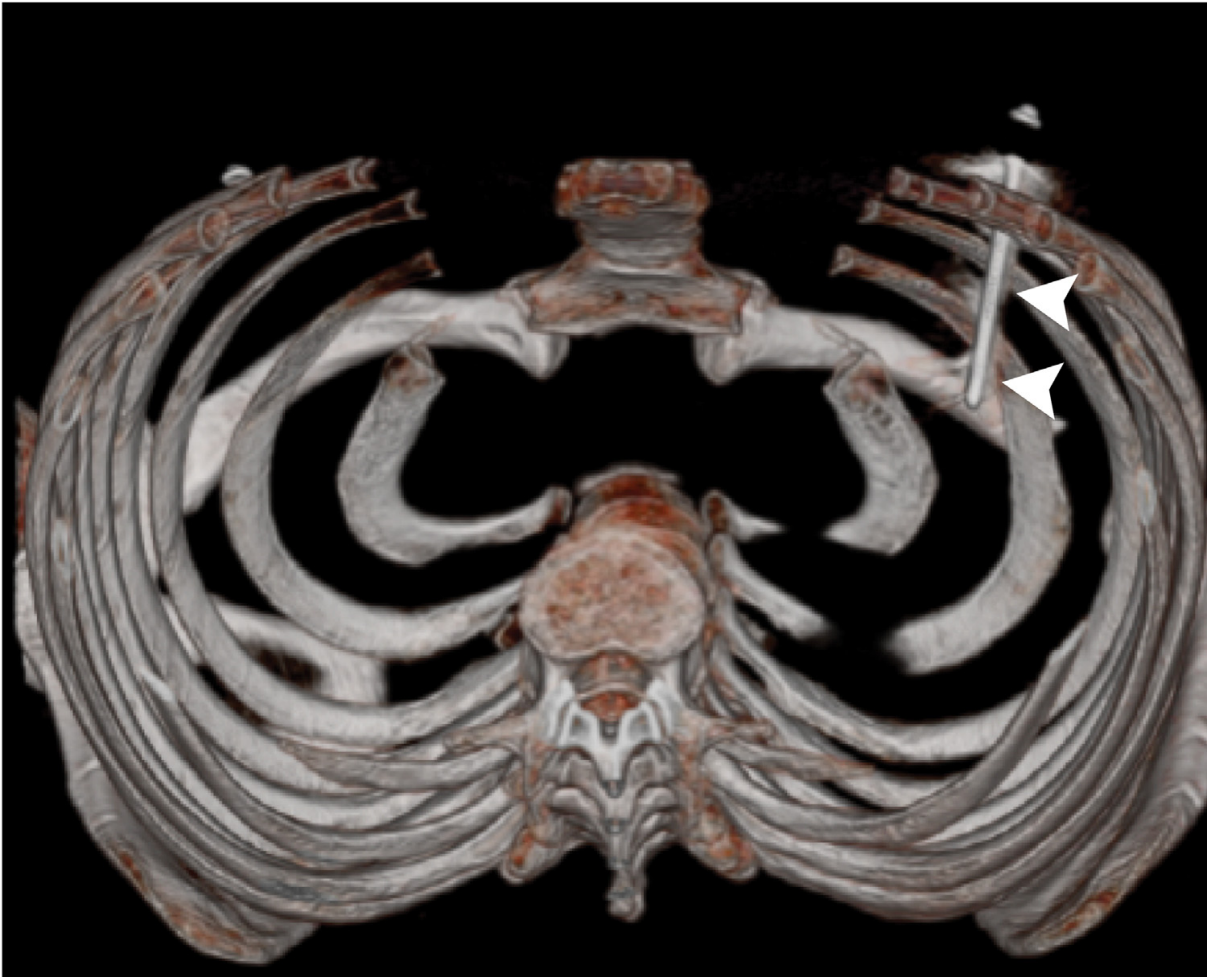
Reconstructed view of the chest CT (inferior view), the nail and its trajectory has been illustrated in silver color (white arrowheads).

**Fig. 7:**
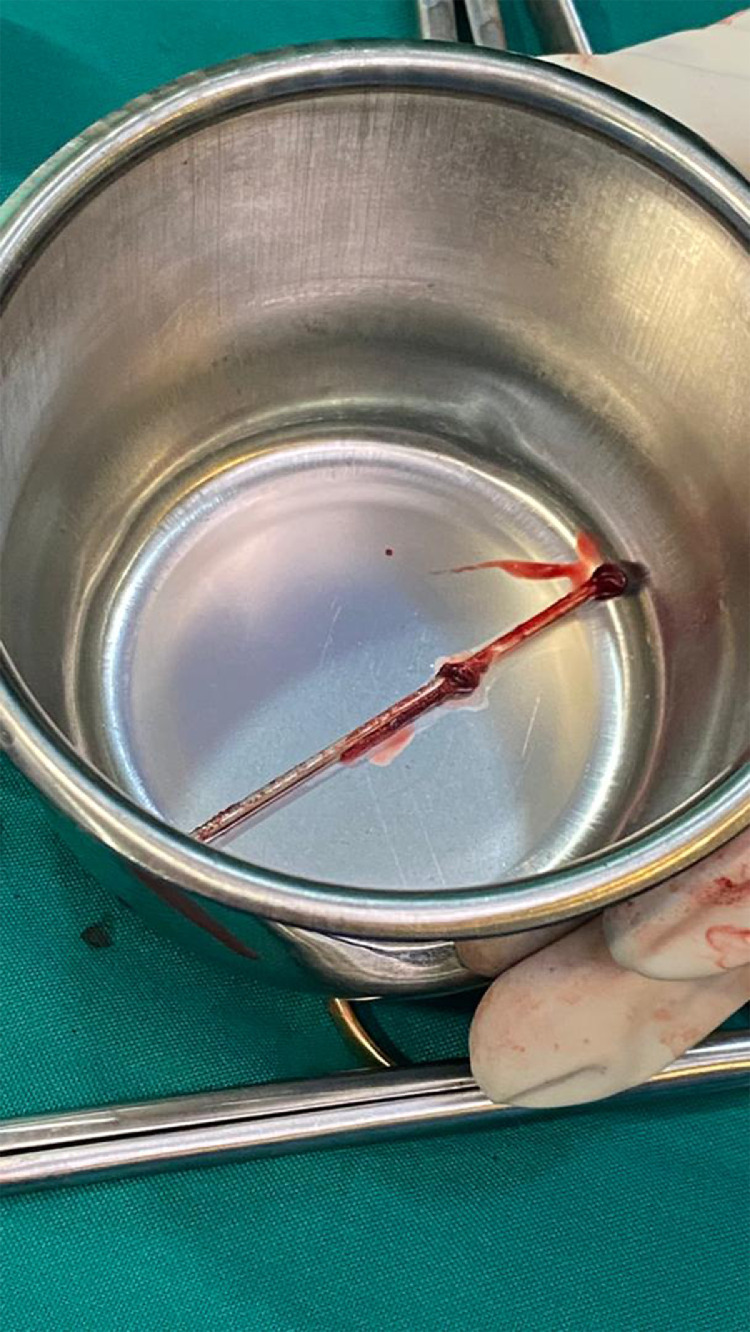
The nail was TIPO type with 44 mm length and a 2.7-mm round head.

A 28-year-old man without previous medical history was brought to the emergency department of the hospital with worsening dyspnea, chest pain, and coughing developed following penetrating chest trauma by a nail gun which happened *5 hours earlier*. He was repairing a sofa at his home using this gun when a nail was misfired to his chest accidentally. Upon primary survey, the patient's airway was intact, he was tachypneic, and had reduced lung sound at left hemithorax. He had a blood pressure of 110/70 mm Hg and heart rate of 94 beats per minute and his heart sounds were muffled but regular S1-S2 were barely heard. He had a GCS score of 15/15. Upon chest inspection, a clean circular puncture wound was noted in the third intercostal space of the left hemithorax at the midclavicular line (Fig. 1). According to the hemodynamic stability, he underwent chest CT scan and it revealed a nail as a sharp hyperdense foreign body that penetrated the chest wall and passed through the lower lobe of the left lung and then entered the pericardium, and finally the anterior aspect of the left ventricle (LV) reaching LV cavity. Also, patchy ground glass opacities at left lower lobe was noted which pertained to hemorrhage (Fig. 2, Fig. 3, Fig. 4, Fig. 5, Fig. 6). Transthoracic echocardiography was performed and chest CT scan observation was confirmed and revealed pericardial effusion. Considering clinical and imaging findings, the patient underwent midline sternotomy. After opening the pericardium, the gush of blood was completely evacuated and controlled by Teflon felt-supported suture and to impede the left anterior descending artery involvement, Teflon-felt wrapping repair was performed in horizontal mattress manner (Video-2). After cardiac suturing, the nail was extracted from the left lung, and the chest wall *(*Fig. 7*)*. Regarding surgery assessment, transesophageal echocardiography was done and confirmed no evidence of further intraventricular dissection, ventricular septal rupture or any residual defect. There was no postoperative complication and he was extubated on postoperative day 2 and the drain output progressively diminished and was removed serially. The patient was discharged 4 day's after admission. His outpatient follow-up, 2 weeks after surgery was uncomplicated.

## Discussion

Chest is the third most common anatomic region for trauma and considering penetrating mechanism, it has approximately 40% mortality. Although, according to previous studies, nail gun injury has less mortality rate (25%), its rarity makes its diagnosis crucial [1,3,4]. Therefore, early vigilant clinical assessment of the patients, and employment of imaging modalities among those with hemodynamic stability, can play a key role in the precise characterization of the injury especially considering rare causes like nail gun injury. Although bedside transthoracic echocardiography can detect cardiac injury including pericardial effusion, cardiac tamponade, valvular involvement, atrial and ventricular dysfunction accurately, its utility to further localize trajectory of the insulting foreign body and estimating the extent of injury in the surrounding anatomic structures is limited. Consequently, chest CT scan can be used to define a spectrum of injuries comprising lungs contusion, aortic artery transection and other vessels injury, pneumothorax, pneumopericardium, pleural and pericardial effusions, pericardial or myocardial lacerations and cardiac luxation. In addition, it is eligible to demonstrate wound track of penetrating trauma and retained foreign bodies as shown in this case [1]. Even though cardiac nail gun injury more commonly involves the right ventricle because of its anterior location to the chest wall, in this case, LV was mainly involved which made it even rarer [3]. This fact highlighted the role of chest CT scan in his management more prominently.

As a result, based on a review of the literature and our observation in the management of this case, prompt utilization of chest CT scan is feasible, particularly when confronting an

unusual presentation of a patient with penetrating chest trauma who is hemodynamically stable to evaluate the traumatic injury and patient's condition more accurately.

*Video-1 legend:* A 28 seconds video showing route of the nail in mediastinum through the left lung and its removal from the lung.

*Video-2 legend:* A 90 seconds video showing Teflon-felt wrapping repair performed in horizontal mattress manner in order to impede the left anterior descending artery involvement.

## Appendix Supplementary materials

Image, video 1

Image, video 2
